# Mechanical Complications of Hip and Knee Spacers Are Common

**DOI:** 10.7759/cureus.38496

**Published:** 2023-05-03

**Authors:** James Costanzo, Joseph McCahon, Anthony T Tokarski, Carl Deirmengian, Tiffany Bridges, Brian E Fliegel, Gregory K Deirmengian

**Affiliations:** 1 Orthopaedic Surgery, Christiana Care Health System, Wilmington, USA; 2 Orthopaedic Surgery, Jefferson Health New Jersey, Stratford, USA; 3 Orthopaedic Surgery, Rothman Orthopaedic Institute, Philadelphia, USA

**Keywords:** cement spacer techniques, cement spacer, revision hip and knee surgery, hip and knee replacement, periprosthetic joint infection

## Abstract

Introduction

Two-stage revision is frequently used for the treatment of periprosthetic joint infection (PJI). Because antibiotic-loaded cement spacers are constructed and implanted as temporary devices, mechanical complications are possible. The purpose of our study was to define the incidence of such mechanical complications, determine associated risk factors, and establish if such complications influence the subsequent success of PJI treatment.

Methods

We identified patients who received an antibiotic spacer for the treatment of PJI at a single center over a six-year timeframe. Medical records and all radiographs were collected and reviewed. Radiographic changes over time were recorded, and mechanical complications were noted. We used multivariate logistic regression analysis to assess risk factors for mechanical spacer complications and assess whether such complications influence the likelihood of subsequent reimplantation and ultimate component retention.

Results

A total of 236 patients were included in the study. There were 82 hip spacers (28% dynamic and 72% static) with a mechanical complication rate of 8.5% and 154 knee spacers (44% dynamic and 56% static) with a mechanical complication rate of 18.2%. Knee spacers were significantly more likely to have mechanical complications than hip spacers. Other risk factors for mechanical complications included bone loss and elevated body mass index (BMI). Bone loss and advanced age were found to be independent risk factors for failure to undergo second-stage reimplantation. Mechanical spacer failure was not an independent risk factor for the likelihood of subsequent reimplantation or ultimate component retention.

Conclusions

Mechanical complications of antibiotic spacers are common but do not appear to negatively impact the likelihood of subsequent reimplantation or component retention. In knee spacers and in patients with bone loss or elevated BMI, appropriate patient counseling and strategies to prevent such complications are recommended.

## Introduction

The mainstay treatment for chronic periprosthetic joint infection (PJI) in the United States is a two-stage revision arthroplasty, with the first stage consisting of implant extraction and placement of an antibiotic-impregnated cement spacer [1]. In conjunction with systemic antibiotics, two-stage revision can successfully treat PJIs with eradication rates as high as 91% [1,2]. Nevertheless, PJI remains one of the most devastating complications of hip and knee arthroplasty.

Despite its success, complications can occur following two-stage revision. Bone loss, joint stiffness, wound complications, recurrence or persistence of the infection, spacer fracture or dislocation, and side effects of local or systemic antibiotics have been reported [3-9]. Nevertheless, spacers perform several important functions such as distributing highly concentrated antibiotics to a localized area and maintaining the joint space and soft tissue tension for future component reimplantation [1,10]. Mechanical complications such as spacer fracture or dislocation have been described in the literature [9,11]. However, the risk factors and frequency of mechanical complications are not well-established.

The purpose of this study is to determine the incidence of mechanical complications of static and dynamic hip and knee spacers at a single institution, the risk factors for mechanical complications, and whether a mechanical complication is an independent risk factor for failure to perform second-stage reimplantation of permanent arthroplasty components.

## Materials and methods

Following institutional review board (IRB) approval (#08R.20) from Thomas Jefferson University Hospital, a retrospective review was performed for all patients who underwent antibiotic spacer placement by one of six fellowship-trained adult reconstruction surgeons at a single institution for hip or knee PJI from 2012 to 2017. All patients who were followed for at least one year following their last surgical procedure were included in the study. Electronic medical records and radiographs were reviewed for all patients meeting the inclusion criteria. Patients without immediate postoperative radiographs or radiographs at least four weeks following spacer implantation were excluded from the analysis. Additionally, patients with prior revision surgery or two-stage exchange were excluded.

A total of 327 patients who underwent hip or knee antibiotic spacer placement were identified. Of these, 91 patients were excluded due to incomplete radiographs (69 patients), initial spacer placement at an outside institution (21 patients), and an above-knee amputation two days following spacer implantation secondary to a vascular injury (one patient). Charts for the resulting 236 cases were retrospectively reviewed for preoperative patient demographics, including age, gender, body mass index (BMI), and Charlson Comorbidity Index (CCI) [12]. Operative reports and immediate postoperative radiographs were reviewed to determine the type of spacer (static versus dynamic), retained hardware in the joint, and any hardware implanted with the spacer.

All antibiotic spacers were fashioned with 2-3 g tobramycin and 2-3 g vancomycin per bag (40 g) of Palacos cement. Static hip spacers consisted of a sphere of cement within the acetabulum and a dowel of cement within the femoral canal. In some cases, at the discretion of the surgeon, a Steinman pin was added to the femoral dowel. Dynamic hip spacers were fashioned using commercially available spacer molds and subsequently press-fit in the femur. Postoperatively, patients were allowed to ambulate using a walker with touchdown weight-bearing until reimplantation.

Static knee spacers were placed in the joint space and allowed to harden with the extremity placed in full extension with gentle manual traction. Alternatively, dynamic knee spacers were fashioned using commercially available spacer molds and then loosely cemented to the distal femur and proximal tibia. Postoperatively, static knee spacer patients were immobilized in full extension and restricted to touchdown weight-bearing until reimplantation. Dynamic knee spacer patients were allowed active assisted motion as tolerated and made protected weight-bearing with a rolling walker until reimplantation.

All patients received six weeks of intravenous antibiotics. After a minimum four-week “drug holiday,” patients were clinically examined and evaluated for persistent infection using the inflammatory markers C-reactive protein (CRP) and erythrocyte sedimentation rate (ESR) and underwent joint aspiration. All patients without evidence of persistent infection underwent reimplantation. If preoperative workup and/or intraoperative findings were consistent with persistent infection, repeat irrigation and debridement, and spacer exchange were performed.

All radiographs were viewed using IDS7 software (Spectra AB, Linköping, Sweden). Immediate postoperative radiographs were reviewed to quantify the amount of bone loss, which was classified as mild or moderate/severe. Hips with greater than Paprosky I acetabular and/or femoral bone loss and knees with greater than Anderson Orthopaedic Research Institute (AORI) type 1 bone loss were considered moderate/severe [13,14].

Follow-up radiographs taken at least four weeks postoperatively were reviewed for mechanical complications including any component breakage, joint or component dislocation, hardware cutout, or periprosthetic fracture. Minor joint or component subluxation or translation was not considered mechanical complications. In addition, mechanical complications that changed the timing or technique of reimplantation were documented. The timing of reimplantation and any subsequent reoperations were also noted.

Statistical analysis was performed using R 3.02 statistical software (R Foundation for Statistical Computing, Vienna, Austria). Multivariate logistic regression analysis was used to assess risk factors for mechanical complications and the likelihood of subsequent reimplantation and final component retention.

## Results

A total of 236 antibiotic spacers were included in the study (Table 1), consisting of 82 hip spacers (59 static spacers (72%) and 23 dynamic spacers (28%)) and 154 knee spacers (68 static spacers (44%) and 86 dynamic spacers (56%)) (Table 2).

**Table 1:** Demographics of the patient cohort

	Total	Hips	Knees
Number	236	82	154
Age (years)	66 (38-95)	63 (38-88)	67 (41-95)
Male	137	44	63
Female	129	38	91
Body mass index	31.6 (17-64)	30.5 (18-64)	32.2 (17-61)
Charlson Comorbidity Index	1.05 (0-8)	0.87 (0-8)	1.14 (0-8)

**Table 2:** Spacer type of the patient cohort

	Hip	Knee
Total	82	154
Dynamic	23 (28%)	68 (44%)
Static	59 (72%)	86 (56%)

In total, 140 (59.3%) patients had mild bone loss and 95 (40.3%) had moderate/severe bone loss.

**Figure 1:**
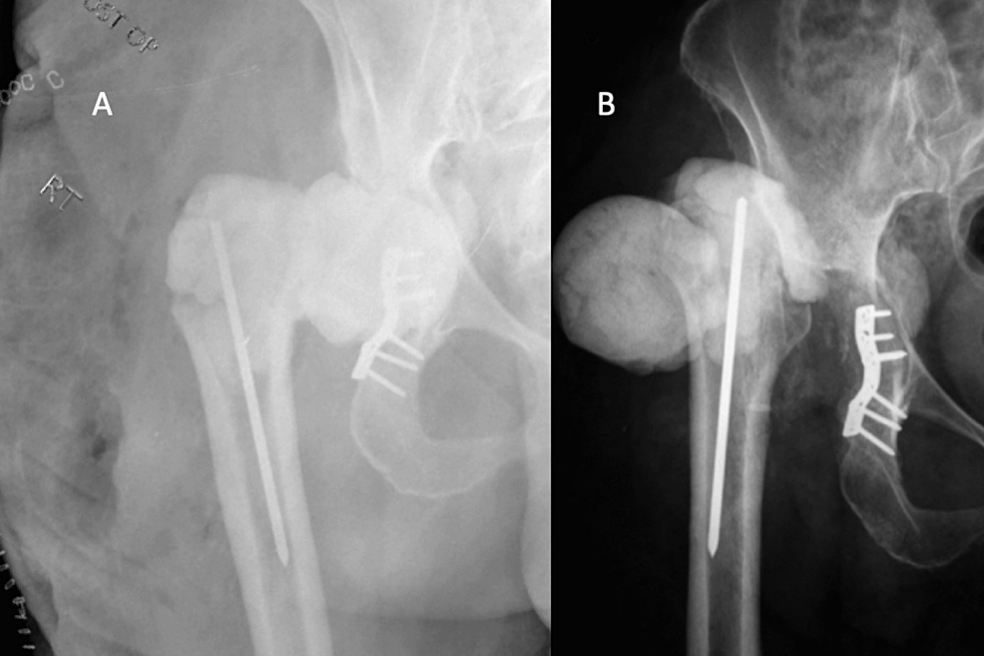
Static hip spacer mechanical complication Immediate postoperative (A) and three-week postoperative (B) anteroposterior radiographs of the hip demonstrating complete dislodgment of the acetabular component and subluxation of the femoral component

There were five (6.1%) mechanical complications identified for hip spacers with all of these occurring in static spacers (8.5%). Complications consisted of one femoral component and four acetabular component dislodgements (Figure 1).

Mechanical complications did not change or hasten reimplantation plans.

**Figure 2:**
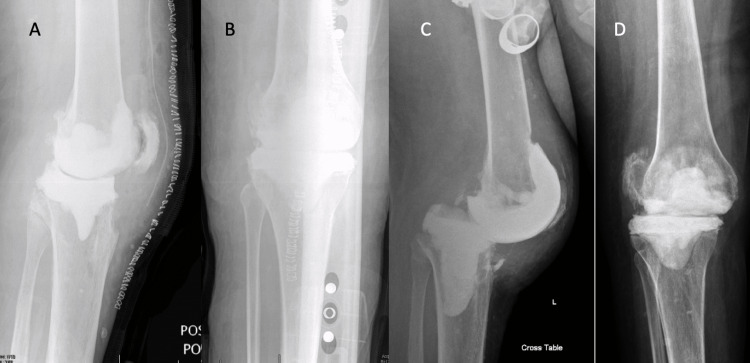
Dynamic knee spacer mechanical complication Immediate postoperative lateral (A) and anteroposterior (B) and six-week postoperative lateral (C) and anteroposterior (D) radiographs demonstrating posterior dislocation of the tibia

There were 28 (18.2%) mechanical complications identified for knee spacers, occurring in 18 (20.9%) static knee spacers and 10 (14.7%) dynamic knee spacers. In static spacers, there were 10 anterior tibial-sided cement dislodgements, five anterior femoral-sided cement dislodgements, and three cases of hardware cutout. In dynamic spacers, there were six anterior joint dislocations, three anterior tibial component dislodgements, and one posterior tibial component dislocation (Figure 2).

Time to knee arthroplasty reimplantation was performed earlier than planned in eight (28.6%) cases due to mechanical complications.

Knee spacers (28/154, 18.2%) were significantly more likely to have a mechanical complication than hip spacers (5/82, 6.1%) (p<0.001) (Table 3).

**Table 3:** Risk factors for complications *Indicates statistical significance

	Mechanical complication	No mechanical complication	p value
Hip	5 (6.1%)	77 (93.9%)	<0.001*
Knee	28 (18.2%)	126 (81.8%)	
Mild bone loss	15 (10.7%)	125 (89.3%)	<0.001*
Moderate/severe bone loss	37 (38.9%)	58 (61.1%)	
Body mass index > 40	12 (42.9%)	16 (57.1%)	0.013*
Body mass index < 40	37 (19.1%)	156 (80.9%)	

Mechanical complications were also significantly higher in patients with moderate/severe bone loss (38.9% versus 10.7%, p<0.001) and BMI > 40 (42.8% versus 19.2%, p=0.013). Spacer hardware was not a significant risk factor for mechanical complications (22% versus 22.2%).

A total of 201 (85.2%) patients proceeded with reimplantation using standard components. The remaining patients failed to undergo reimplantation at the latest follow-up and, instead, either did not pursue surgery, received treatment for infection without subsequent reimplantation, died, or were lost to follow-up. Of the 201 reimplanted patients, 172 (85.6%) did not demonstrate subsequent evidence of recurrent or persistent infection at the latest follow-up. The remaining 29 (14.4%) patients were diagnosed with a recurrent or persistent infection based on Musculoskeletal Infection Society Criteria. Based on multivariate analysis, a mechanical complication of spacers was not an independent risk factor for failure to undergo reimplantation or PJI recurrence. Alternatively, moderate/severe bone loss (p=0.007) and advanced age (p=0.037) were significant risk factors for failure to undergo reimplantation.

## Discussion

Two-stage revision is the preferred method of treatment for chronic PJI in North America [1]. This study presents the results of a two-stage exchange at a single institution with a focus on spacer mechanical complications.

Although complications of hip and knee spacers are well-known, there is a paucity of literature regarding risk factors for mechanical complications and how these complications may impact the ultimate treatment outcome for PJI [3-5,15-20]. Jacobs et al. [21] demonstrated a 7% dislocation and 2% fracture rate in dynamic hip spacers and no mechanical complications following static spacers. Interestingly, we found mechanical complications in static hip spacers only, in complete contrast to the previously reported numbers.

In our study, knee spacers were significantly more likely to have mechanical complications than hip spacers (18.2% versus 6.1%, p<0.001). This may be due to differences in bending movement and bony fixation causing greater forces across the antibiotic spacer and bone-spacer interface in the knee. Difficulty with patient compliance with the use of a brace and limiting weight-bearing may also contribute to the difference in complications at the knee and hip. Increased mechanical complication rates were also noted in patients with BMI > 40 and in patients with moderate/severe bone loss.

Our study did not demonstrate a statistically significant difference between mechanical complications in static versus articulating spacers. Hip spacers trended toward more mechanical complications in static spacers versus dynamic spacers, but this difference was not statistically significant. Previous studies have shown some benefits to dynamic spacers, including range of motion, with comparable rates of infection control. There remains, however, no definitive difference among overall outcomes or mechanical complications [22-24]. In a large systematic review and meta-analysis, Fiore et al. [23] reported an improved range of motion with articulating versus dynamic spacers, but no difference in reinfection rates, complications, and clinical scores. Their meta-analysis demonstrated no significant difference regarding the incidence of non-infection-related complications between articulating and static spacers.

There is no consensus in the literature regarding the use of hardware within cement spacers. Hypothetical advantages of improved stability may be offset by providing an additional surface for bacterial adherence and biofilm formation. In a retrospective comparative study of posterior-stabilized articulating spacers, Lin et al. [25] reported no spacer subluxations or dislocations in mechanically reinforced spacers compared to those without Kirschner wire-reinforced cams. There have also been techniques described in the hip to improve the mechanical stability of articulating spacers via a hybrid screw-cement fixation technique [26]. Our study did not demonstrate a difference in mechanical complications between spacers with or without hardware. Given the relatively small subgroup of patients with hardware in this study, further investigation into this subject is warranted.

The ultimate consequences of mechanical complications have not been previously analyzed. In our study, mechanical complications following hip spacers did not alter subsequent treatment plans. In knee spacers, however, reimplantation of permanent components was hastened in nearly a third of patients with mechanical complications. This may be attributed to differences in the soft tissue envelope in the hip as compared to the knee. The relatively thinner soft tissue of the knee is less likely to tolerate significant changes in the position of the components and the joint, which may lead surgeons to alter surgical plans to protect both the skin and the vascularity of the knee. Despite this difference between hips and knees, no patient failed to undergo reimplantation secondary to a mechanical complication in our cohort.

Our study has several limitations that are mostly inherent to its retrospective nature. First, it is possible that patients with complications may have followed up at an outside institution, which may lead to an underestimation of complications. Second, multiple surgeons performed the procedures, and therefore, surgical techniques and postoperative protocols were not completely standardized. Despite this, all surgeons were fellowship-trained arthroplasty surgeons, and the large number of cases should serve to limit the effects of heterogeneity among surgeons. Third, there was an unequal ratio of knees and hips as well as dynamic and static spacers. However, these subgroups were analyzed separately to attempt to identify differences within each group. Lastly, the long-term follow-up of patients was limited, which may have led to the underestimation of the rate of recurrent/persistent infection in our cohort.

## Conclusions

Mechanical complications of antibiotic spacers are common. This study does not demonstrate a negative effect on the outcome of treatment of PJI secondary to mechanical complications. Despite this, a mechanical complication remains a potentially painful and distressing event for the patient and may trigger the surgeon to alter carefully designed treatment plans. Because of this, strategies to prevent such complications are suggested, especially in patients with significant bone loss or elevated BMI.
